# PLGA-based dual targeted nanoparticles enhance miRNA transfection efficiency in hepatic carcinoma

**DOI:** 10.1038/srep46250

**Published:** 2017-04-07

**Authors:** Chenlei Cai, Yuexia Xie, liangliang Wu, Xiaojing Chen, Hongmei Liu, Yan Zhou, Hanbing Zou, Dejun Liu, Yanan Zhao, Xianming Kong, Peifeng Liu

**Affiliations:** 1 Central Laboratory, Ren Ji Hospital, School of Medicine, Shanghai Jiao Tong University, Shanghai, 200127, China; 2 State Key Laboratory of Oncogenes and Related Genes, Shanghai Cancer Institute, Ren Ji Hospital, School of Medicine, Shanghai Jiao Tong University, Shanghai, 200032, China; 3 Department of Biliary-Pancreatic Surgery, Ren Ji Hospital, School of Medicine, Shanghai Jiao Tong University, Shanghai, 200127, China

## Abstract

Hepatic carcinoma (HCC) is a lethal disease associated with high morbidity and poor prognosis. Recently years, gene therapies have offered novel modalities to improve the prognosis of HCC patients. MicroRNA-99a (miR-99a) is frequently down-regulated in HCC, where it acts as a tumor suppressor. Therefore, we constructed monomethoxy (polyethylene glycol)-poly(D,L-lactide-*co*-glycolide)-poly(L-lysine)-lactobionic acid- anti-vascular endothelial growth factor antibody (mPEG-PLGA-PLL-LA/VEGFab or PEAL-LA/VEGFab) nanoparticles (NPs) with highly specific targeting properties as carriers to restore the expression of miR-99a both *in vitro* and *in vivo*, to inhibit HCC progression. *In vitro*, PEAL-LA/VEGFab NPs showed more efficient delivery of miR-99a to HepG2 cells than the conventional transfection reagent Lipofectamine^TM^2000 (Lip2000). The higher delivery efficiency associated with PEAL-LA/VEGFab NPs consequently resulted in down-regulation of target genes and suppression of the proliferation, migration and invasion of HepG2 cells. *In vivo*, miR-99a-PEAL-LA/VEGFab NPs inhibited tumor xenograft growth in HCC-bearing mice without causing obvious systemic toxicity. Our results demonstrate that PEAL-LA/VEGFab NPs selectively and effectively deliver miR-99a to HCC cells based on the double-targeting character of these nanoparticles, thereby offering potential for translation into effective clinical therapies for HCC.

Hepatocellular carcinoma (HCC) is one of the most common and lethal cancers, accounting for nearly 1 million deaths every year worldwide^1^,^2^,^3^,^4^,^5^,^6^. Owing to the development of resistance of HCC to traditional radiation or chemotherapy regimens^6^,^7^,^8^, surgical resection is the preferred treatment^2^. Unfortunately, most HCC patients are diagnosed at advanced stages, which severely limits the success of surgical therapy^6^. Therefore, finding novel effective treatments for HCC is of great importance. MicroRNAs (miRNAs) are endogenous, small non-coding RNAs that regulate target gene expression by post-transcriptional repression^9^. Growing evidence demonstrates the involvement of miRNAs in tumor development and that deregulation of miRNAs could contribute to carcinogenesis in several tumors including HCC^10^,^11^. For instance, Hou *et al*. found that miR-199a/b-3p, the third most highly expressed miRNA in normal liver, markedly decreased in HCC and its decrement significantly associates with poor prognosis of HCC patients^12^,^13^,^14^. As the sixth most abundant microRNA in normal human liver tissue, microRNA-99a (miR-99a) is markedly down-regulated in hepatocellular carcinoma tissues and cell lines, which significantly correlates with poor prognosis of HCC patients^12^,^13^. Restoration of miR-99a not only dramatically suppresses HCC cell growth, migration and invasion *in vitro* but also efficiently inhibits tumor growth *in vivo* by inhibiting mammalian target of rapamycin (mTOR) and Argonaute2(Ago2) signaling, which suggests that miR-99a could be used in targeted HCC treatments^13^,^15^.

Despite the potential efficacy of miR-99a in HCC therapy, intrinsic issues with respect to the large molecular weight and hydrophilic nature encumbers the ability of RNA-based drugs, such as miR-99a, to enter cells through passive diffusion^16^,^17^. In addition, rapid enzymolysis in the plasma is another hurdle preventing systemic delivery of naked miRNAs to the intended sites *in vivo*^16^,^17^. These challenges have provoked the development of a long-lasting and efficient delivery system for miRNAs. Although some viral and non-viral vectors have been used with relatively higher delivery efficacy, these options are limited by their potential toxicity, high cost and/or non-selective nature of those vectors, especially *in vivo*^18^,^19^,^20^,^21^.

To address this issue, we constructed a dual-targeted delivery system using monomethoxy (polyethylene glycol)-poly(D,L-lactide-*co*-glycolide)-poly(L-lysine)-lactobionic acid- anti-vascular endothelial growth factor antibody (mPEG-PLGA-PLL-LA/VEGFab or PEAL-LA/VEGFab), which offers multiple advantages, including improved biocompatibility, stability and cost efficiency as well as negligible toxicity and high selectivity toward tumor cells. We show experimental evidence that PEAL-LA/VEGFab nanoparticles can be used to deliver miR-99a specifically to the cancer site to effectively treat HCC.

In this constructed delivery system consists of PLGA, mPEG, PLL, LA and VEGFab, in which PLGA is an FDA-approved material that has been widely used in clinical applications to encapsulate drugs or genes efficiently through hydrophobic interactions^22^,^23^,^24^. The mPEG-modification improves the stability and prolongs the circulation half-lives of the nanoparticles (NPs)^25^. Moreover, PLL, which shows low toxicity, contains several primary amine groups that can absorb negatively charged miRNAs through electrostatic interactions^25^,^26^. The targeting moiety LA specifically binds the asialoglycoprotein receptor, which is a membrane protein abundantly expressed on the surfaces of hepatocellular carcinoma cells^27^,^28^,^29^,^30^. Another targeting moiety, anti-vascular endothelial growth factor antibody (VEGFab), is capable of specifically bind VEGF, which is an endothelial cell-specific marker highly expressed in the majority of human tumors, including HCC^31^,^32^,^33^,^34^,^35^. LA and VEGFab were covalently conjugated to PEAL NPs to improve the targeting of PEAL-LA/VEGFab NPs to HCC. Our work achieves *in vitro* and vivo treatment of HCC with excellent therapeutic efficacy using PEAL-LA/VEGFab NPs, and our work highlights the promise of miR-99a-PEAL-LA/VEGFab NPs for use in HCC therapy.

## Results and Discussion

### Characterization of the NPs

**Figure 1:**
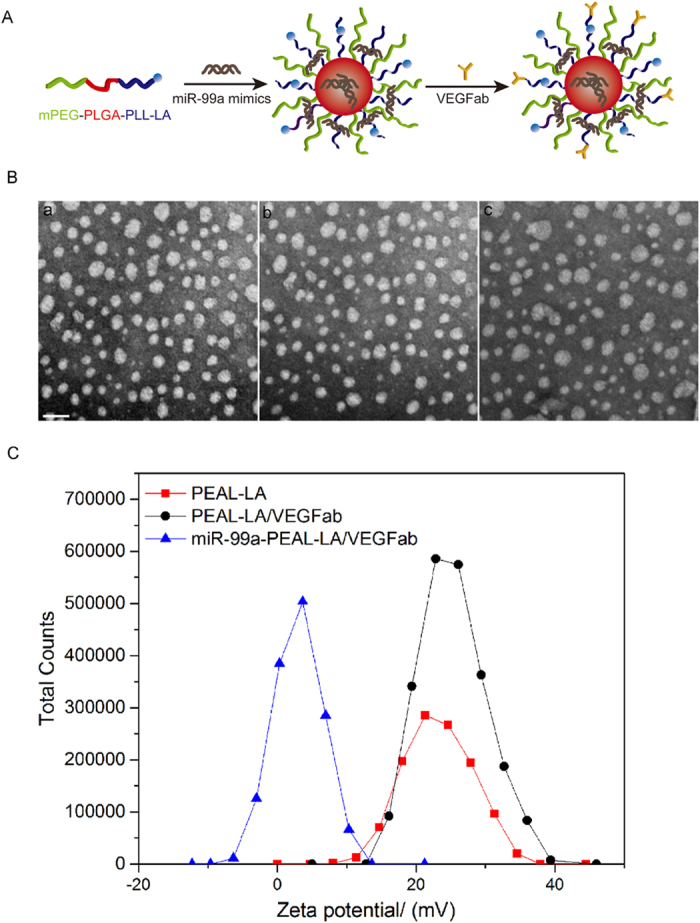
(**A**) Synthesis of miR-99a-PEAL-LA/VEGFab micellar nanoparticles. (**B**) TEM images of PEAL-LA NPs (a), PEAL-LA/VEGFab NPs (b) and miR-99a-PEAL-LA/VEGFab NPs (c). The scale bar is 100 nm. (**C**) Zeta potential images of PEAL-LA NPs, PEAL-LA/VEGFab NPs and miR-99a-PEAL-LA/VEGFab NPs.

In the present work, we prepared the miR-99a-PEAL-LA/VEGFab NPs using a two-step synthetic route (Fig. 1A). First, the miR-99a was loaded onto the PEAL-LA copolymers via ultrasonic emulsification to form miR-99a-PEAL-LA NPs. Second, VEGFab was conjugated to the miR-99a-PEAL-LA NPs to form miR-99a-PEAL-LA/VEGFab NPs. The as-synthesized polymers formed a micellar structure in aqueous solution with a hydrophobic PLGA core, hydrophilic mPEG and positively charged PLL chains. The miR-99a was entrapped in the hydrophobic PLGA core via hydrophobic interactions, and partial miR-99a adsorbed to the positively charged PLL chains through electrostatic interactions.

The morphology of the NPs was characterized by transmission electron microscopy (TEM). The monodispersed PEAL-LA NPs, PEAL-LA/VEGFab NPs and miR-99a-PEAL-LA/VEGFab NPs exhibited similar spherical morphologies with average diameters of 50.06 ± 1.16 nm, 49.46 ± 1.19 nm and 50.95 ± 1.21 nm, respectively (Fig. 1B). Zeta potentials of the NPs were further analyzed through dynamic light scattering (DLS). Due to the presence of protonated amine groups in the PLL, both PEAL-LA NPs and PEAL-LA/VEGFab NPs were positively charged with average zeta potentials of 23.2 mV and 25.3 mV (Fig. 1C), respectively. With the encapsulation of negatively charged miR-99a, the average zeta potential of miR-99a-PEAL-LA/VEGFab NPs decreased to 3.02 mV, which is indicative of the effective loading of the miR-99a. Physicochemical properties, including the size, morphology and charge of the NPs, are highly important, as they can directly affect the stability, cellular uptake and biodistribution of nanoparticles. The miR-99a-PEAL-LA/VEGFab NPs we constructed showed a relatively uniform size distribution with an average diameter of approximately 50 nm, which is an optimal size for NPs as non-viral vectors for the following reasons. First, the miR-99a-PEAL-LA/VEGFab NPs are small enough to pass through the cell membrane by receptor-mediated endocytosis and escape uptake by the reticuloendothelial system (NPs > 100 nm are recognized by the RES)^16^,^36^. Second, our NPs are large enough to avoid renal elimination (NPs < 6 nm are eliminated by the kidneys)^37^,^38^, which increases NPs retention time. Moreover, the nearly neutral charge of miR-99a-PEAL-LA/VEGFab NPs should favor binding with the target receptor on the cell membrane and promote NPs internalization.

In addition, miR-99a-PEAL-LA/VEGFab NPs exhibited a remarkably sustained release of miR-99a. Both miR-99a-PEAL-LA NPs and miR-99a-PEAL-LA/VEGFab NPs showed rapid miR-99a release of approximately 30% at 12 h. Over the subsequent 132 h, the miR-99a was sustainably released to final cumulative levels of 79.01 ± 2.08% and 81.33 ± 1.86% for miR-99a-PEAL-LA NPs and miR-99a-PEAL-LA/VEGFab NPs, respectively, which contributes to prolonged interaction time between miR-99a and the target genes and enhances therapeutic efficiency.

### Cytotoxicity of the NPs

**Figure 2:**
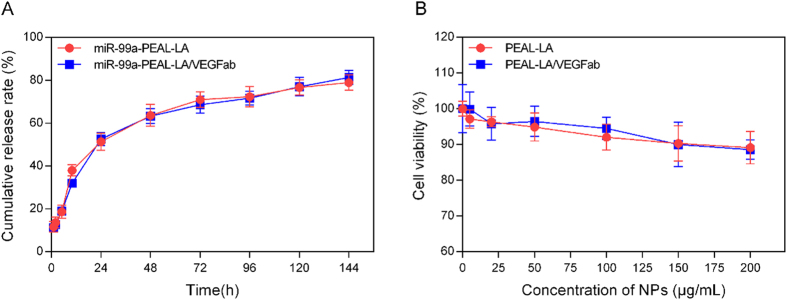
(**A**) *In vitro* release properties of miR-99a-PEAL-LA NPs and miR-99a-mPEG-PEAL-LA/VEGFab NPs at 37 °C; Data are presented as the means ± S.D. (n = 3). (**B**) *In vitro* cytotoxicity of PEAL-LA NPs and PEAL-LA-VEGFab NPs after incubation with HepG2 cells for 24 h. Data are presented as the means ± S.D. (n = 6).

We next tested the *in vitro* toxicity of NPs in HepG2 cells using the CCK-8 assay. We observed no obvious changes in cell viability in cells treated with either PEAL-LA NPs or PEAL-LA/VEGFab NPs compared with cells treated with PBS after a 24-h incubation (Fig. 2B). Even with exposure to higher NP concentrations (200 μg/mL), more than 80% of cells remained viable, which suggests that both PEAL-LA NPs and PEAL-LA/VEGFab NPs exert negligible toxicity and are highly compatible with living cells.

### CLSM analysis

**Figure 3:**
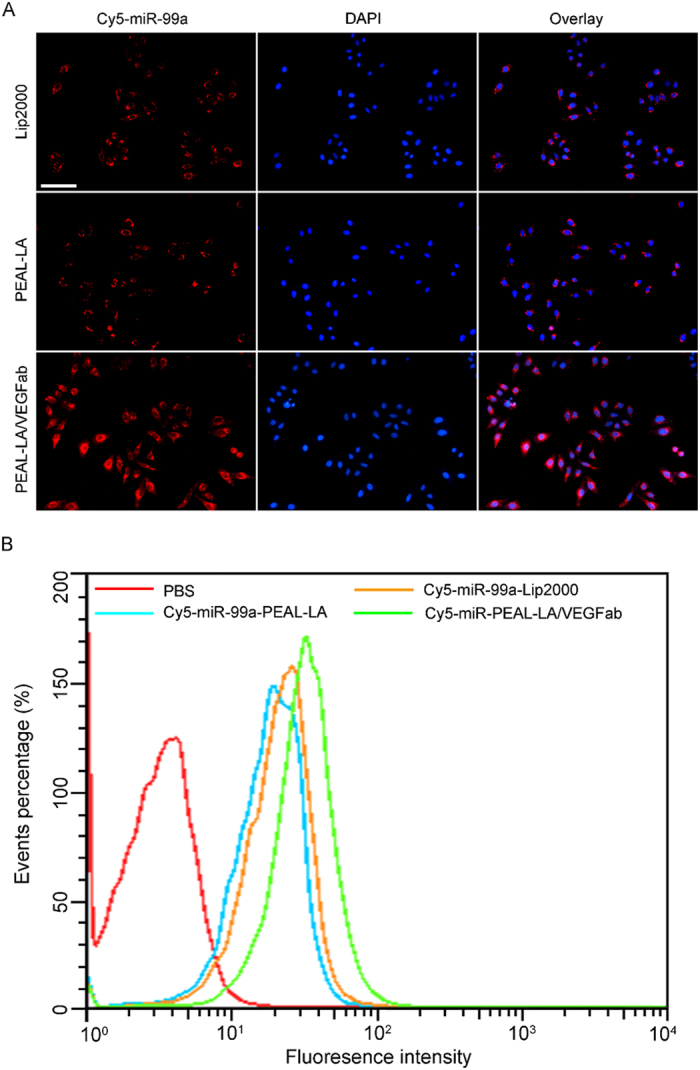
(**A**) CLSM images of HepG2 cells incubated with Cy5(red)-miR-99a-Lip2000, Cy5-miR-99a-PEAL-LA NPs or Cy5-miR-99a-PEAL-LA/VEGFab NPs for 4 h. Magnification 200 ×, the scale bar is 100 μm. (**B**) Flow cytometry analysis of HepG2 cells treated with Cy5-miR-99a-Lip2000, Cy5-miR-99a-PEAL-LA NPs or Cy5-miR-99a-PEAL-LA/VEGFab NPs for 4 hours.

CLSM analysis was conducted to evaluate the delivery efficiency of miR-99a by NPs. To facilitate observations by CLSM, miR-99a was labeled with the Cy5 fluorescent probe to form Cy5-miR-99a-PEAL-LA NPs and Cy5-miR-99a-PEAL-LA/VEGFab NPs. The commercial transfection reagent Lipofectamine^TM^2000 (Lip2000) was used as a control. As shown in Fig. 3A, after 4 h of co-incubation, the fluorescent signal (red) in cells treated with Cy5-miR-99a-PEAL-LA NPs was similar to those incubated with Cy5-miR-Lip2000, suggesting that PEAL-LA NPs can achieve similar transfection efficiencies to Lip2000. Furthermore, cells cultured with Cy5-miR-99a-PEAL-LA/VEGFab NPs exhibited much brighter fluorescence emission than those cultured with Cy5-miR-99a-PEAL-LA NPs or Cy5-miR-99a-Lip2000, suggesting that Cy5-miR-99a-PEAL-LA/VEGFab NPs containing dual-targeting moieties (LA and VEGFab) exhibit a synergistic targeted effect and show significant enhancement of miR-99a delivery.

### Flow cytometry analysis

Flow cytometry analysis was subsequently performed to quantitatively analyze the cellular uptake of NPs. Figure 3B shows that the fluorescence intensity of cells incubated with Cy5-miR-99a-Lip2000, Cy5-miR-99a-PEAL-LA NPs and Cy5-miR-99a-PEAL-LA/VEGFab NPs increased by 72.1%, 79.7% and 93.3%, respectively, compared with the control group (cells treated with PBS), demonstrating an improved binding ability of PEAL-LA/VEGFab NPs for HepG2 cells. These results are consistent with the CLSM results and further confirm that the modification of double-targeting moieties (LA and VEGFab) results in greater endocytosis of miR-99a-PEAL-LA/VEGFab NPs by HepG2 cells and enhances delivery efficiency of miR-99a.

### Analysis of qRT-PCR and western blot

**Figure 4:**
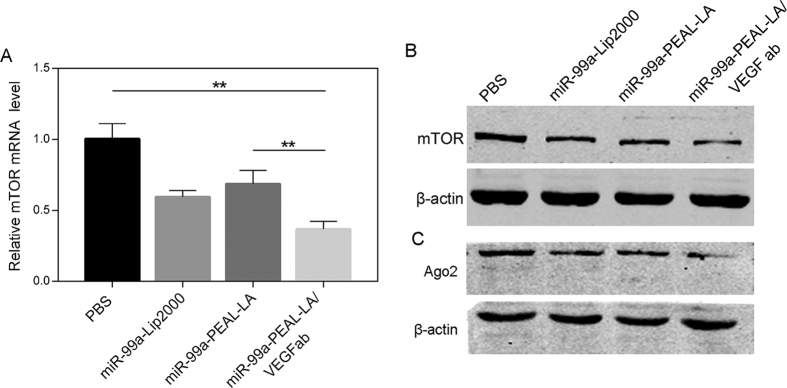
Relative mTOR mRNA (**A**), mTOR protein (**B**) and Ago2 protein (**C**) expression of HepG2 cells treated with PBS, miR-99a-Lip2000, miR-99a-PEAL-LA NPs or miR-99a-PEAL-LA/VEGFab NPs, which were determined by quantitative real-time PCR and western blot analysis. (n = 3), **p < 0.01.

The ideal anticancer therapy would need to effectively facilitate miR-99a interaction with its target genes through the effective delivery of miR-99a to target cells, such as HepG2 hepatocellular carcinoma cells. This delivery should restore miR-99a expression, reduce expression of its target genes and suppress HCC growth. The mTOR signaling pathway is closely related with tumorigenesis and its down-regulation could suppress tumor progression^13^,^14^. Previous research has revealed that mTOR was an acknowledged target gene of miR-99a and the restoration of miR-99a in HCC could dramatically reduce its expression on both mRNA and protein level^13^. In addition, Argonaute-2 (Ago2) is a member of Argonaute gene family that function as a catalytic component of RNA-induced silencing complex (RISC) to cleave the target messenger RNAs (mRNAs) that are complementary to the guiding small interference RNAs (siRNAs) or microRNAs (miRNAs). Previous study showed that Ago2 was a novel target gene of miR-99a. The overexpression of miR-99a could significantly inhibit the expression of Ago2 protein, whereas the mRNA level of Ago2 was not significantly reduced, suggesting that miR-99a might inhibit Ago2 through translation repression rather than mRNA degradation^15^. Therefore, if the miR-99a was restored effectively in HCC, the expression of mTOR mRNA, mTOR protein as well as Ago2 protein would be inhibited significantly. To test the efficacy of the NPs-mediated delivery of miR-99a, we analyzed HepG2 cells treated with our NPs-miR-99a nanocomplexes for the expression of mTOR mRNA by qRT-PCR, mTOR protein and Ago2 protein by western blot. As shown in Fig. 4A, decreased expression of mTOR mRNA was observed in cells treated with miR-99a-Lip2000, miR-99a-PEAL-LA NPs and miR-99a-PEAL-LA/VEGFab NPs compared with the PBS group. Notably, miR-99a-PEAL-LA/VEGFab NPs showed the strongest inhibitory effect on mTOR mRNA expression, as mTOR mRNA expression was decreased by more than 60%. Analysis of mTOR protein expression by western blot revealed knock-down efficiency consistent with the mRNA expression data. Down-regulation of mTOR protein expression was most pronounced when miR-99a-PEAL-LA/VEGFab NPs were used (Fig. 4B), which correlated with the inhibition of tumor progression. In addition, as to the Ago2 protein (Fig. 4C), compared with the PBS group, decreased expression of Ago2 protein was observed in cells treated with miR-99a-Lip2000, miR-99a-PEAL-LA NPs and miR-99a-PEAL-LA/VEGFab NPs and among them, miR-99a-PEAL-LA/VEGFab NPs showed the most pronounced inhibitory effect resulting in more than 60% decrease of Ago2 protein expression. The western blot results revealed that the PEAL-LA/VEGFab NPs could mediate the most effectively delivery of miR-99a and subsequently regulate the expression of Ago2 protein.

### Clonogenic assay

**Figure 5:**
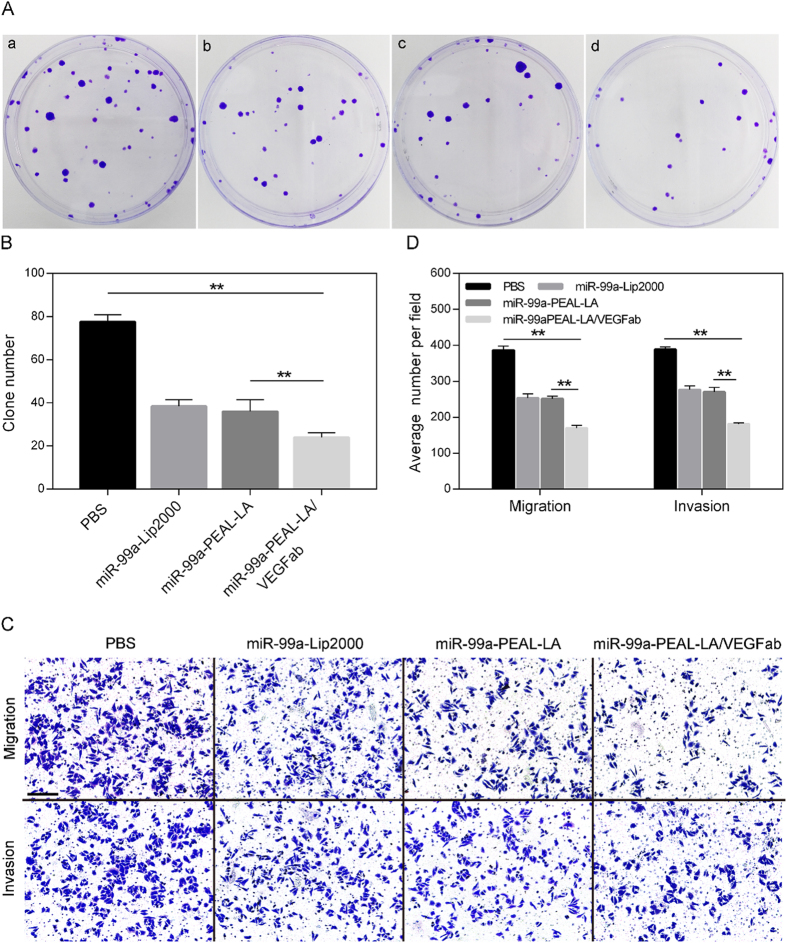
(**A**) The effects on colony formation of HepG2 cells treated with PBS (a), miR-99a-Lip2000 (b), miR-99a-PEAL-LA NPs (c) and miR-99a-PEAL-LA-VEGFab NPs (d) was evaluated by the clonogenic assay. (**B**) The corresponding quantification of colony formation efficiency. Data are presented as the means ± S.D., (n = 5). **p < 0.01. (**C**) Inhibition of migration and invasion of HepG2 cells upon treatment with PBS, miR-99a-Lip2000, miR-99a-PEAL-LA NPs and miR-99a-PEAL-LA/VEGFab NPs. Data are represented as the means ± S.D. (N = 3). Magnification 100 ×, the scale bar is 200 μm. (**D**) The corresponding quantification of migration and invasion. (n = 3) **p < 0.01.

Next, we evaluated the ability of NPs to inhibit tumor progression *in vitro*. First, the clonogenic assay was performed to investigate the inhibitory effect of the NPs on clonogenic formation in HepG2 cells. As shown in Fig. 5A and B, a reduced number of colonies was observed in cells treated with miR-99a-Lip2000 and miR-99a-PEAL-LA NPs compared with the control group (38.40 ± 1.36 *vs.* 77.60 ± 1.44, *P* < 0.01; 36.00 ± 2.43 *vs*. 77.60 ± 1.44, *P* < 0.01). Moreover, more effective inhibition was observed in cells treated with miR-99a-PEAL-LA/VEGFab NPs (24.00 ± 1.00 *vs.* 77.60 ± 1.43, *P* < 0.01), which exhibited an obviously higher inhibition rate of 69.1% for clonogenic growth potential in HCC. These results suggest that the dual-targeting PEAL-LA/VEGFab NPs could deliver miR-99a into HepG2 cells most effectively, which results in the inhibition of the proliferation of tumor cells and enhanced anti-tumor activity *in vitro*.

### Migration and invasion assays *in vitro*

As key phenotypic characteristics of tumor cells, migration and invasion greatly influence the prognosis of tumors. To explore the effect of NPs on the migratory and invasive capability of HepG2 cells, transwell assays using transwell chambers with (invasion assay) or without (migration assay) Matrigel coating were performed, the results of which are shown in Fig. 5C and D. Although miR-99a-Lip2000 or miR-99a-PEAL-LA NPs could suppress the migration of HepG2 cells, as reflected by the reduced number of migrating cells (254.00 ± 6.56 *vs.* 386.30 ± 6.77, *P* < 0.01; 251.70 ± 4.10 *vs.* 386.30 ± 6.77, *P* < 0.01), their inhibitory efficiency was far less than that of miR-99a-PEAL-LA/VEGFab NPs, which resulted in a substantial decrease in the migratory activity of HepG2 cells (170.00 ± 4.51 *vs.* 386.30 ± 6.77, *P* < 0.01). Similarly, the results of the invasion assay depicted in Fig. 5C and D also showed that miR-99a-PEAL-LA/VEGFab NPs achieve better inhibition of cell invasion than PBS, miR-99a-Lip2000, or miR-99a-PEAL-LA NPs (207.30 ± 4.67 *vs.* 388.30 ± 4.18 *vs.* 276.70 ± 5.78 *vs.* 270.30 ± 7.36), thereby inhibiting tumor metastasis and enhancing the therapeutic effect.

### Anticancer efficiency

**Figure 6:**
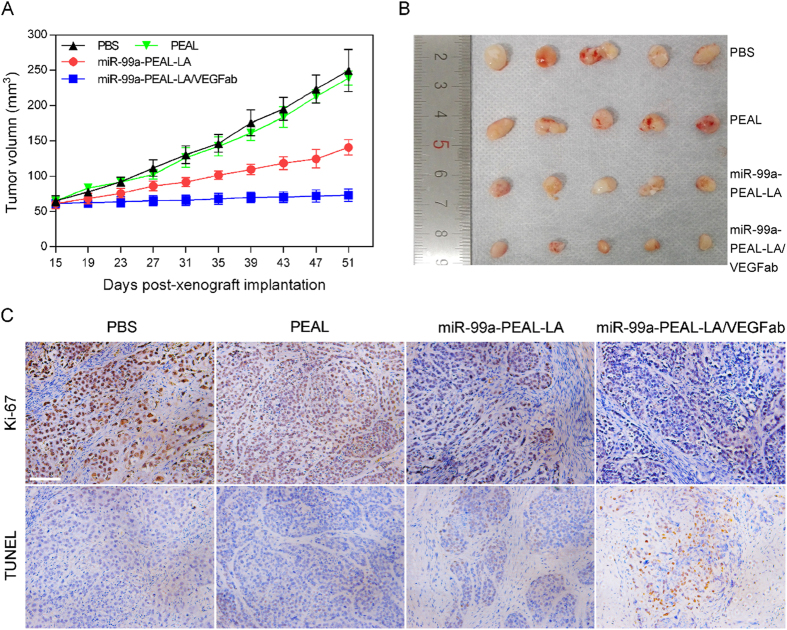
Intravenous delivery of miR-99a-loaded NPs suppresses growth of HCC xenografts in nude mice. (**A**) Relative tumor volume of nude mice bearing HCC tumors treated with PBS, blank PEAL NPs, miR-99a-PEAL-LA NPs and miR-99a-PEAL-LA/VEGFab NPs. Data are presented as the means ± S.D. (n = 5). (**B**) Photographs of excised tumors from the different groups harvested at the endpoint of treatment. (**C**) Ki-67 analysis by immunohistochemistry and TUNEL assays of tumor tissues after treatment with the various formulations. The scale bar is 200 μm.

To explore the *in vivo* antitumor efficiency of NPs, we used a HepG2 subcutaneous xenograft nude mouse model to monitor tumor progression and test the superiority of the miR-99-PEAL-LA/VEGFab NPs delivery system. The NPs were injected through the tail vein, which is consistent with the anticipated clinical route of administration. As indicated in Fig. 6A and B, we observed no significant difference in tumor size among mice treated with PBS or blank PEAL NPs alone. In contrast, the tumor growth inhibition of mice treated with miR-99a-PEAL-LA NPs or miR-99a-PEAL-LA-VEGFab NPs was much higher than that of mice receiving PBS or PEAL NPs. The final tumor size of mice treated with miR-99a-PEAL-LA/VEGFab NPs was approximately 50 mm^3^, which was remarkably smaller than that of the other groups, confirming effective antitumor efficiency of this NP formulation in HCC-bearing mice. These results were further validated by immunohistochemistry and TUNEL assay analysis (Fig. 6C). The expression of Ki-67, a representative marker for proliferating cells, was detected to investigate the proliferative activity of HCC xenografts treated with NPs. The percentage of Ki-67-positive cells was remarkably decreased in the miR-99a-PEAL-LA/VEGFab group compared with the other groups (Fig. 6C). These data are consistent with the results of the TUNEL assay, in which apoptosis induced by miR-99a-PEAL-LA/VEGFab NPs treatment was increased compared with that of either PBS, PEAL NP or miR-99a-PEAL-LA NPs treatment (Fig. 6C). These results suggest that miR-99a-PEAL-LA/VEGFab NPs effectively suppress tumor growth by inhibiting proliferation and inducing apoptosis in HCC tumor cells.

**Figure 7:**
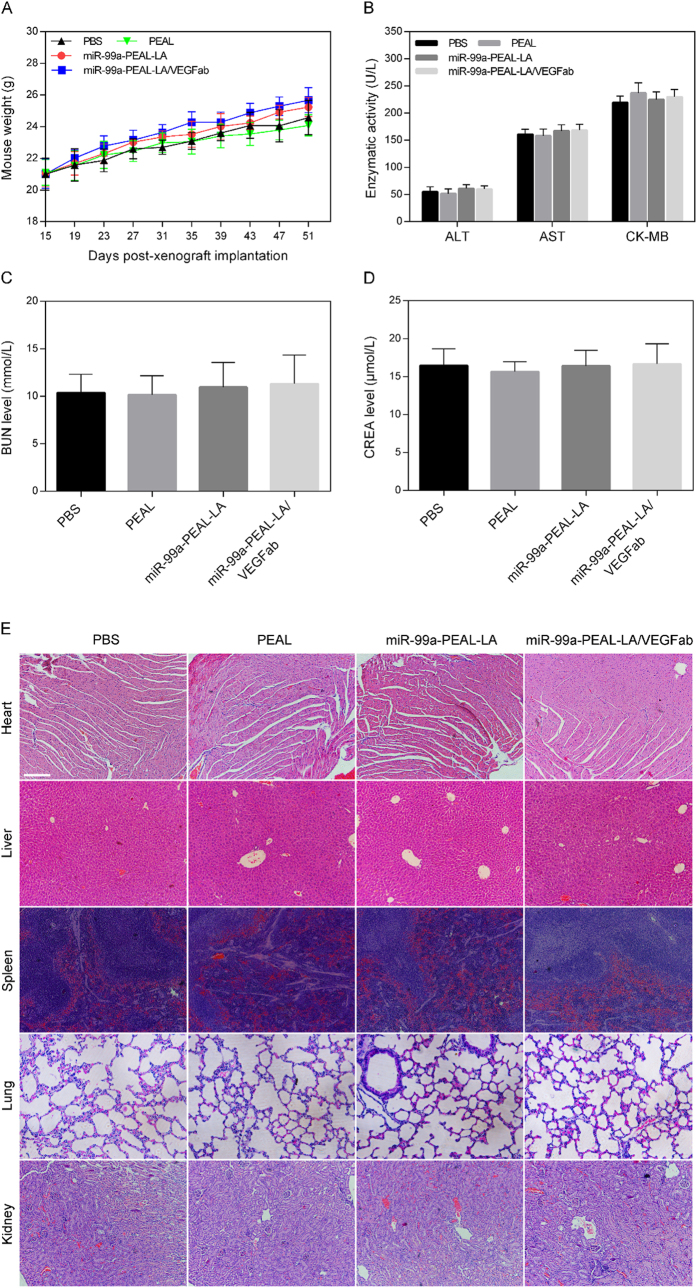
*In vivo* toxicity analysis of HCC-bearing nude mice treated with the various formulations. (**A**) Body weight changes of mice receiving various treatments. (**B**,**C** and **D**) Represent the blood biochemistry evaluation of heart, liver and kidney functions of mice treated with the different formulations. Data are represented as the means ± S.D. (n = 5). (**E**) Hematoxylin and eosin (HE) staining of organs, including heart, liver, spleen, lung and kidney from mice treated with the various formulations. The scale bar is 200 μm.

Finally, the potential in vivo toxicity of miR-99a-PEAL-LA/VEGFab NPs was further investigated. We observed the behaviors and weight change of the mice receiving various treatments throughout the treatment period and harvested their blood and organs (heart, liver, spleen, lung and kidneys) at the endpoint for serum chemistry analysis and hematoxylin and eosin (HE) staining. We observed no apparent signs of toxic response and minor weight changes in the mice for all experimental groups over the treatment period (Fig. 7A). A blood biochemistry analysis reflecting the function of major organs was conducted for all the mice at the end of treatment. As shown in Fig. 7B–D, all biochemistry parameters of mice treated with miR-99a-PEAL-LA/VEGFab NPs were within ranges similar to mice treated with PBS. HE staining results shown in Fig. 7E demonstrated that no obvious histopathological abnormalities or tissue damage was noticed in mice receiving treatment in all experimental groups. These results suggest that the miR-99a-PEAL-LA/VEGFab NPs induce no significant systemic toxicity or other physiological complications *in vivo* at the tested dose. However, further studies are still needed to systematically evaluate the potential toxicity of miR-99a-PEAL-LA/VEGFab NPs at a range of doses.

Overall, miR-99a-PEAL-LA/VEGFab NPs were successfully constructed and applied as a highly effective HCC treatment modality. Double-targeted miR-99a-PEAL-LA/VEGFab NPs had excellent specificity for HepG2 hepatocellular carcinoma cells and exhibited synergic efficacy in delivering miR-99a to the target cells. The miR-99a delivery resulted in the suppression of mTOR expression and led to effective inhibition of the migration, invasion and clonogenic formation of HepG2 hepatocellular carcinoma cells. *In vivo* antitumor experiments further verified the effectiveness of miR-99a-PEAL-LA/VEGFab NPs as a therapeutic nanomedicine. Furthermore, no noticeable toxic responses *in vitro* or *in vivo* were observed at the tested doses. Although our study is focused on the HCC therapy by miR-99a-loaded nanocarriers, due to the high similarity with human internal environment, these miRNA-based anti-cancer therapies may also be effective for other tumors such as renal cell carcinoma and breast cancer^39^,^40^. This study highlights the great potential of miR-99a-PEAL-LA/VEGFab NPs for tumor therapy applications.

## Methods

### Materials

Monomethoxy (polyethylene glycol) (mPEG) (Mn = 2000) was obtained from Sigma-Aldrich (St. Louis, MO, USA). D,L-lactide and glycolide were purchased from GLACO (Beijing, China). Catalyst stannous octoate [Sn(OCt)_2_] was purchased from the Zhixing Chemical Co. Ltd. (Shanghai, China). N^ε^-(carbonylbenzoxy)-L-lysine and N-t-butoxycarbonyl-L-phenylalanine (Boc-L-Phe) were purchased from GL Biochem Co. Ltd (Shanghai, China). Nε-(Z)-lysine-N-carboxyanhydride (NCA) was synthesized and purified using the method reported by Dorman *et al*.^41^. Lactobionic acid (LA) and dimethyl sulfoxide (DMSO) were purchased from Sigma-Aldrich (St. Louis, MO, USA). Pluronic-F68 was obtained from BASF (Ludwigshafen, Germany). 1-(3-Dimethylaminopropyl)-3-ethylcarbodiimide hydrochloride (EDC) was obtained from Aladdin (Shanghai, China). N-hydroxysuccinimide sodium salt (NHS) was obtained from Sinopharm Chemical Reagent Co. Ltd. (Shanghai, China). Dulbecco’s Modified Eagle Medium (DMEM), 10,000 units/mL penicillin, 10 mg/mL streptomycin, fetal bovine serum (FBS) and 0.25% ethylene diamine tetraacetic acid (EDTA)-trypsin were obtained from Gibco, Life Technologies (Grand Island, USA). A cell counting kit-8 (CCK-8) was purchased from Dojindo (Kumamoto, Japan). A monoclonal antibody against mammalian target of rapamycin (anti-mTOR) was purchased from Cell Signaling Technology (Danvers, MA, USA). The monoclonal antibody against Ago2 (anti-Ago2) was obtained from Abcam (Cambridge, UK). The monoclonal antibody against vascular endothelial growth factor (anti-VEGF/VEGFab) was purchased from Santa Cruz Biotechnology (CA, USA). The anti-β-actin antibody was purchased from HuaAn Biotechnology (Zhejiang, China). M-PER mammalian protein extraction reagent and BCA protein assay kits were purchased from Thermo Scientific (Rockford, lL, USA). TRIzol Reagent was purchased from Ambion, Life Technologies (Grand Island, USA). Lipofectamine^TM^2000 (Lip2000) was obtained from Invitrogen, Life Technologies (Grand Island, USA). A PrimeScript^TM^ RT reagent kit with gDNA Eraser and SYBR Premix Ex Taq^TM^ were purchased from Takara (Tokyo, Japan); 2-(4-Amidinophenyl)-6-indolecarbamidine dihydrochloride (DAPI) was obtained from the Beyotime Institute of Biotechnology (Haimen, China). Has-miR-99a mimics (miR-99a, sense strand, 5′-AACCCGUAGAUCCGAUCUUGUG-3′ and anti-sense strand, 5′-CAAGAUCGGAUCUACGGGUUUU-3′), Cy5 labeled has-miR-99a mimics (Cy5-miR-99a) were purchased from Ribobio (Guangzhou, China). Hepatic carcinoma cell line HepG2 was purchased from the cell bank of the Chinese Academy of Sciences (Shanghai, China). Cells were cultured in DMEM containing 10% fetal bovine serum (FBS) and 1% antibiotics (100 U/mL penicillin and 100 μg/mL streptomycin) in a humidified atmosphere containing 5% CO_2_ at 37 °C. Transwell chambers were purchased from Corning (Bedford, MA, USA). Nude BALB/c mice (4 to 5 weeks old, male, average body weight: 17.72 ± 0.34 g) were supplied by Shanghai Experimental Animal Centre of the Chinese Academy of Sciences (Shanghai, China). All animal experiments were performed in accordance with the National Institutes of Health Guide for the Care and Use of Laboratory Animals and with the approval of the Animal Care and Use Committee of Shanghai Jiao Tong University.

### Synthesis of mPEG-PLGA-PLL-LA (PEAL-LA)

The PEAL-LA copolymers were synthesized according to a recently reported method from us^42^,^43^,^44^, and the detailed steps were as follows: (1) Synthesis of hydroxyl-terminated mPEG-PLGA copolymer through Sn(OCt)_2_-catalyzed ring-opening polymerization (ROP) of glycolide and D,L-lactide, which was initiated by mPEG. (2) Synthesis of Boc-L-Phe end-capped mPEG-PLGA polymer by converting Boc-Phe to hydroxyl-terminated mPEG-PLGA copolymer. (3) Synthesis of amino-terminated mPEG-PLGA by removing the t-butoxycarbonyl end-group from the Boc-L-Phe end-capped mPEG-PLGA polymer. (4) Synthesis of mPEG-PLGA-b-poly (Nε-(Z)-L-lysine) polymer by ROP of the NCA, which was initiated by the amino-terminated mPEG-PLGA. (5) Synthesis of amino-terminated PEAL by removing the Nε-(carbonylbenzoxy) end-group from the mPEG-PLGA-poly (Nε-(Z)-L-lysine) polymer. (6) Synthesis of PEAL-LA (PEAL: LA = 1:1 mol:mol) by conjugating LA to the amino-terminated PEAL using EDC and NHS.

### Preparation of miR-99a-PEAL-LA NPs

One nanomoles of miR-99a was dissolved in 50 μL of RNase-free water and emulsified in 500 μL dichloromethane solution containing 5 mg of PEAL-LA copolymer by sonication (Scientz sonicator probe, Scientz, China) at 400 W for 1 min to obtain a water/oil emulsion. Then, 2 mL of Pluronic^TM^ F68 water solution (1 mg/mL) was added to the water/oil emulsion and sonicated again to obtain a water/oil/water emulsion. The resulting water/oil/water emulsion was transferred to a rotary evaporator to evaporate the organic solvent and obtain the miR-99a-PEAL-LA NPs.

### Preparation of miR-99a-PEAL-LA/VEGFab NPs

After synthesis of the nanoparticles, 250 μL of EDC (1 mg/mL) and 250 μL of NHS (1 mg/mL) were added to a 5 mL solution containing 10 mg of miR-99a-PEAL-LANPs. Then, VEGFab was added to the solution, and the mixture was agitated on a magnetic stir plate at room temperature for 4 h. Subsequently, the solution was centrifuged (15,000 rpm, 4 °C, 20 min) to remove the unreacted reagents and unconjugated antibody. The obtained sediment was washed and then re-dissolved in PBS to obtain miR-99a-PEAL-LA/VEGFab NPs.

### Characterization of NPs

The morphology of the NPs was characterized by transmission electron microscopy (TEM) using a Hitachi H-7000 TEM operated at an acceleration voltage of 200 kV. The zeta potentials of the NPs were assessed by dynamic light scattering (DLS) (Malvern, Instruments, UK).

### MicroR-99a release from the NPs

Two milliliters of miR-99a-PEAL-LA NPs or miR-99a-PEAL-LA/VEGFab NPs solution was dispensed in an RNase-free tube containing 5 mL of TE buffer, and the tubes were shaken horizontally in a thermostatic shaker (37 °C, 120 rpm/min). Then, the miR-99a-NPs suspension was subjected to centrifugation (15,000 rpm, 4 °C, 30 min) at predetermined intervals. The deposit was re-suspended in fresh TE buffer for re-incubation, and the supernatant was collected and subjected to spectrophotometric analysis at 260 nm using an ultraviolet-visible spectrophotometer.

### Cytotoxicity of the NPs

The CCK-8 assay was used to evaluate the cytotoxicity of the PEAL-LA NPs and PEAL-LA/VEGFab NPs. In brief, 2 × 10^4^ HepG2 cells/well were seeded in a 96-well-plate (Corning, NY, USA) and incubated overnight. The NPs were added to the wells at concentrations ranging from 5 μg/mL to 200 μg/mL and incubated for 24 h. Then, 10 μl of CCK-8 water solution was added to the wells, and the plate was further incubated at 37 °C for 1 h. Finally, the absorbance was measured at 450 nm using a microplate reader.

### Confocal laser scanning microscopy

Confocal laser scanning microscopy (CLSM) was employed to evaluate the delivery efficiency of miR-99a. Lipofectamine^TM^2000 (Lip2000) transfection reagent was used as a positive control. In brief, HepG2 cells (2 × 10^5^ cells/well) were seeded into a CLSM dish and incubated overnight. Then, Cy5-miR-99a-Lip2000, Cy5-miR-99a-PEAL-LA NPs or Cy5-miR-99a-PEAL-LA/VEGFab NPs, each containing equivalent amounts of Cy5-miR-99a, was added to the culture dish and incubated in the dark for 4 h. Then, the cells were fixed with 4% paraformaldehyde for 15 min and stained with DAPI for another 10 min. The cellular uptake of the Cy5-labeled miR-99a was determined using an FV1000 confocal laser scanning microscope (Leica, Germany).

### Flow cytometry analysis

Flow cytometry was applied to quantitatively evaluate the uptake of miR-99a. In brief, HepG2 cells (5 × 10^5^ cells/well) were seeded in 60-mm dishes and incubated overnight. Then, Cy5-miR-99a-Lip2000, Cy5-miR-99a-PEAL-LA NPs or Cy5-miR-99a-PEAL-LA/VEGFab NPs, each containing an equivalent amount of Cy-5-miR-99a, was added to the cells and incubated for 4 h. Cells treated with PBS were used as a negative control. Then, the cells were washed twice with PBS, trypsinized, centrifuged and re-suspended in 500 μL of PBS. The cellular uptake was measured using a FacsAria II Sorp flow cytometer (Becton Dickinson, USA).

### Quantitative real-time PCR (qRT-PCR/qPCR) analysis

Quantitative real-time PCR was performed as previously described. In brief, HepG2 cells (5 × 10^5^ cells/well) were seeded in 60-mm dishes and incubated overnight. Then, miR-99a-Lip2000, miR-99a-PEAL-LA NPs or miR-99a-PEAL-LA/VEGFab NPs, each containing an equivalent amount of miR-99a, was added and incubated for 6 h. Cells treated with PBS were used as a negative control. Then, the medium was refreshed, and the cells were incubated for another 24 h. Total RNA was isolated from HepG2 cells using TRIzol reagent according to the manufacturer’s recommended protocol (Ambion, Life Technologies). After measuring the RNA concentration using a Nano-Drop 2000 spectrophotometer, 1 μg of RNA was reverse-transcribed into cDNA using an RT Reagent Kit containing gDNA Eraser. Then, the cDNA was subjected to qRT-PCR analysis using SYBR Premix Ex Taq. The two-step qPCR process was conducted on a Roche LightCycler480 as follows: denaturation at 95 °C for 30 s, followed by 45 cycles of PCR at 95 °C for 5 s and 60 °C for 20 s. The housekeeping gene GAPDH was used as an endogenous reference. The relative gene expression values were calculated using the ΔΔCt method. The primers used in the PCR reactions were as follows: mTOR-forward 5′-CGCTGTCATCCCTTTATCG-3′, reverse 5′-ATGCTCAAACACCTCCACC-3′, and GAPDH-forward 5′-GAAGGTGAAGGTCGGAGTC-3′, reverse 5′-GAAGATGGTGATGGGATTTC-3′.

### Western blot analysis

HepG2 cells were seeded and treated as described for the qPCR analysis and were harvested 72 h later. Then, the cells were washed twice with cold PBS and lysed with M-PER mammalian protein extraction reagent supplemented with complete protease inhibitor, PMSF and cocktail. The cell lysates were incubated on ice for 30 min and centrifuged at 16,000 g for 30 min at 4 °C; the supernatants were collected after centrifugation. The BCA Protein Assay Kit was used to determine the protein concentration. An equal amount of protein (30 μg) was separated on 8% bis-Tris-polyacrylamide gels and then transferred (250 mA, 180 min) onto nitrocellulose membranes. After blocking with 5% bovine albumin (BSA) for 1 h, the membranes were incubated with mTOR monoclonal antibody (1:1000) and Ago2 monoclonal antibody (1:1000) overnight at 4 °C followed by incubation with goat anti-rabbit or anti-mouse IgG-HRP secondary antibody (1:10,000) for 1 h at room temperature. After washing with Tris-buffered saline containing 0.1% Tween-20 several times, the protein bands were detected and quantified using an Odyssey infrared imaging system (LI-COR Biosciences).

### Migration and invasion assays *in vitro*

HepG2 cells (3 × 10^5^ cells/well) were seeded in 6-well plates and cultured overnight. Then, miR-99a-Lip2000, miR-99a-PEAL-LA NPs or miR-99a-PEAL-LA/VEGFab NPs, each containing an equivalent amount of miR-99a, was added to cells and co-cultured for 6 h. PBS treatment was used as a negative control. 6 h later, the medium was refreshed, and the cells were incubated for another 48 h. Then, 4 × 10^4^ cells pretreated with the various formulations (PBS, miR-99a-Lip2000, miR-99a-PEAL-LA NPs or miR-99a-PEAL-LA/VEGFab NPs) in serum-free medium were seeded in Transwell chambers (8 μm pore) uncoated (migration assay) or coated with Matrigel (invasion assay), which were then transferred to 24-well plates filled with complete medium as a chemoattractant. After incubation for 24 h, the non-migrating or non-invading cells left on the top of the chamber were removed by scratching. The migrated or invaded cells at the bottom of the membrane were fixed with 4% paraformaldehyde for 10 min, followed by staining with crystal violet for 30 min. The stained cells on the bottom membrane were counted under a microscope (5 fields per membrane).

### Clonogenic assay

HepG2 cells were seeded, treated and harvested as described in the migration and invasion assays. Briefly, 200 HepG2 cells from each group (PBS, miR-99a-Lip2000, miR-99a-PEAL-LA NPs and miR-99a-PEAL-LA/VEGFab NPs) were seeded in 60-mm dishes containing complete medium and were cultured for 14 days at 37 °C in a 5% CO_2_ incubator. Then, the colonies were fixed with 4% paraformaldehyde for 10 min, stained with crystal violet for 30 min and counted.

### Animal experiment

To establish the HCC-bearing mouse models, 7 × 10^6^ HepG2 cells were suspended in 200 μL of PBS and subcutaneously injected into the backs of nude BALB/c mice. When the tumor volume (V; mm^3^) reached approximately 50 mm^3^ (V = length × width^2^/2, measured using a Vernier caliper), the tumor-bearing mice were randomly divided into four groups (five mice per group) and were treated as follows: (i) PBS (in a volume of 0.2 mL), (ii) blank PEAL NPs, (iii) miR-99a-PEAL-LA NPs (at a dose of 0.53 mg/kg miR-99a), (iv) miR-99a-PEAL-LA/VEGFab NPs (at a dose of 0.53 mg/kg miR-99a). All the formulations mentioned above were administered via tail vein injection every 2 days for 1 month. Tumor size and body weight were measured every 4 days. Finally, the mice were sacrificed 7 days after the final injection, and tissues (heart, liver, spleen, lung and kidney) along with blood samples were collected for further analysis.

### Immunohistochemistry (IHC)

Tumor tissues were fully fixed with 4% paraformaldehyde and 4-μm paraffin-embedded sections were prepared for examination. The expression of Ki-67 protein was detected using an avidin-biotin immunohistochemical technique according to the manufacturer’s protocol. Heat-induced epitope retrieval by citric acid buffer at pH 6.0 was performed, and 3% H_2_O_2_ was applied to quench endogenous peroxidase activity. Then, the tumor tissues were incubated with primary Ki-67 antibody at 4 °C overnight followed by incubation with biotin-labeled goat secondary antibody and then with streptavidin–horseradish peroxidase. The reaction was visualized with DAB and counter-stained with hematoxylin. The staining for Ki-67 was mainly localized to the nucleus of proliferating cells. A semiquantitative evaluation method was adopted to evaluate the staining results.

### TUNEL assay

Apoptosis in the tumor sections was analyzed using the terminal deoxyribonucleotidyl transferase (TdT)-mediated dUTP nick-end labeling (TUNEL) assay, which was performed using a commercial TUNEL detection kit (Roche, Switzerland) according to the manufacturer’s suggested protocol. The number of TUNEL-positive cells was counted in 10 randomly selected microscopic fields.

### Statistical analysis

Each experiment was repeated independently at least 3 times. All the data are presented as the means ± standard deviation (S.D.). Student’s t-test was applied to assess statistical significance using Graph Pad Prism 6 software, and *P* < 0.05 was considered statistically significant.

## Additional Information

**How to cite this article**: Cai, C. *et al*. PLGA-based dual targeted nanoparticles enhance miRNA transfection efficiency in hepatic carcinoma. *Sci. Rep.* **7**, 46250; doi: 10.1038/srep46250 (2017).

**Publisher's note:** Springer Nature remains neutral with regard to jurisdictional claims in published maps and institutional affiliations.
