# Author Correction: PLGA-based dual targeted nanoparticles enhance miRNA transfection efficiency in hepatic carcinoma

**DOI:** 10.1038/s41598-020-63754-6

**Published:** 2020-04-20

**Authors:** Chenlei Cai, Yuexia Xie, Liangliang Wu, Xiaojing Chen, Hongmei Liu, Yan Zhou, Hanbing Zou, Dejun Liu, Yanan Zhao, Xianming Kong, Peifeng Liu

**Affiliations:** 1 Central Laboratory, Ren Ji Hospital, School of Medicine, Shanghai Jiao Tong University, Shanghai, 200127 China; 2 State Key Laboratory of Oncogenes and Related Genes, Shanghai Cancer Institute, Ren Ji Hospital, School of Medicine, Shanghai Jiao Tong University, Shanghai, 200032 China; 3 Department of Biliary-Pancreatic Surgery, Ren Ji Hospital, School of Medicine, Shanghai Jiao Tong University, Shanghai, 200127 China

Correction to: *Scientific Reports* 10.1038/srep46250, published online 07 April 2017

This Article contains an error in Figure 1B where the incorrect image was shown for PEAL-LA/VEGFab NPs. The correct Figure 1 appears below.

**Figure 1:**
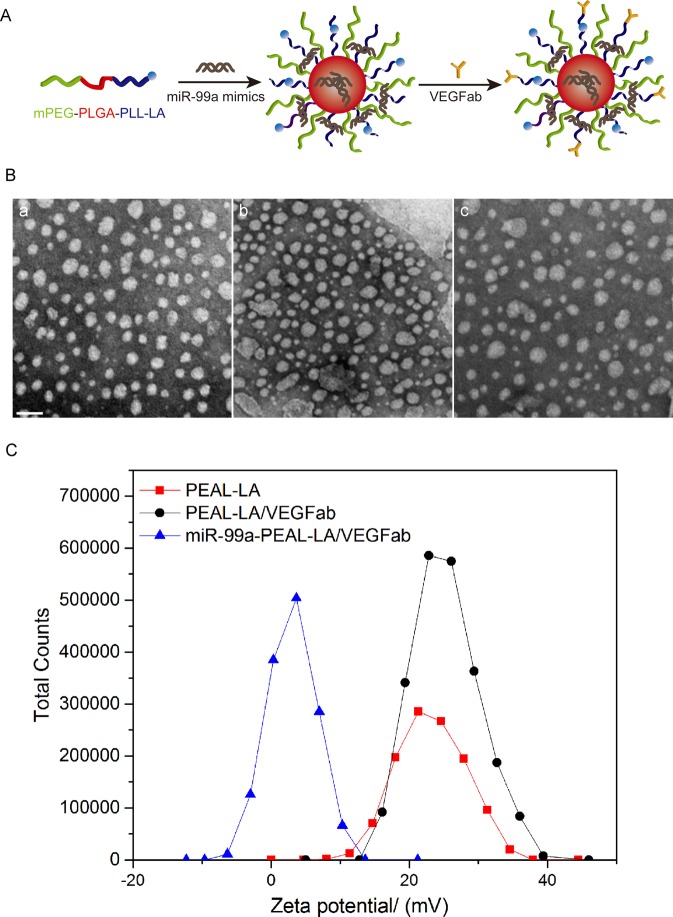
.

